# Roles of Plasmacytoid Dendritic Cells in Gastric Cancer

**DOI:** 10.3389/fonc.2022.818314

**Published:** 2022-03-03

**Authors:** Jinpu Yang, Xia Liu, Yiwen Cheng, Jingchen Zhang, Feng Ji, Zongxin Ling

**Affiliations:** ^1^ Department of Gastroenterology, The First Affiliated Hospital, Zhejiang University School of Medicine, Hangzhou, China; ^2^ Department of Intensive Care Unit, The First Affiliated Hospital, School of Medicine, Zhejiang University, Hangzhou, China; ^3^ Collaborative Innovation Center for Diagnosis and Treatment of Infectious Diseases, State Key Laboratory for Diagnosis and Treatment of Infectious Diseases, National Clinical Research Center for Infectious Diseases, The First Affiliated Hospital, School of Medicine, Zhejiang University, Hangzhou, China; ^4^ Jinan Microecological Biomedicine Shandong Laboratory, Jinan, China

**Keywords:** gastric cancer, *Helicobacter pylori*, immune modulation, plasmacytoid dendritic cells, tumor microenvironment

## Abstract

Gastric cancer (GC) is the fifth most common neoplasm and the third most deadly cancer in humans worldwide. *Helicobacter pylori* infection is the most important causative factor of gastric carcinogenesis, and activates host innate and adaptive immune responses. As key constituents of the tumor immune microenvironment, plasmacytoid dendritic cells (pDCs) are increasingly attracting attention owing to their potential roles in immunosuppression. We recently reported that pDCs have vital roles in the development of immunosuppression in GC. Clarifying the contribution of pDCs to the development and progression of GC may lead to improvements in cancer therapy. In this review, we summarize current knowledge regarding immune modulation in GC, especially the roles of pDCs in GC carcinogenesis and treatment strategies.

## 1 Introduction

Gastric cancer (GC) is one of the most common malignancies worldwide (1). GC has become relatively rare in the United States, but remains common in Asia (2); it is the third most commonly diagnosed cancer (10.6%) and the second most common cause of cancer-related death (13.6%) in China, and thus constitutes a serious health burden on society (3). *Helicobacter pylori* infection, high salt consumption, smoking, low fruit and vegetables consumption, and high alcohol consumption are risk factors for GC (4). As the early phase of GC is asymptomatic or has nonspecific symptoms, most patients are diagnosed at advanced stages. Therefore, effective strategies for early diagnosis, prevention, and treatment are needed.

**Figure 1:**
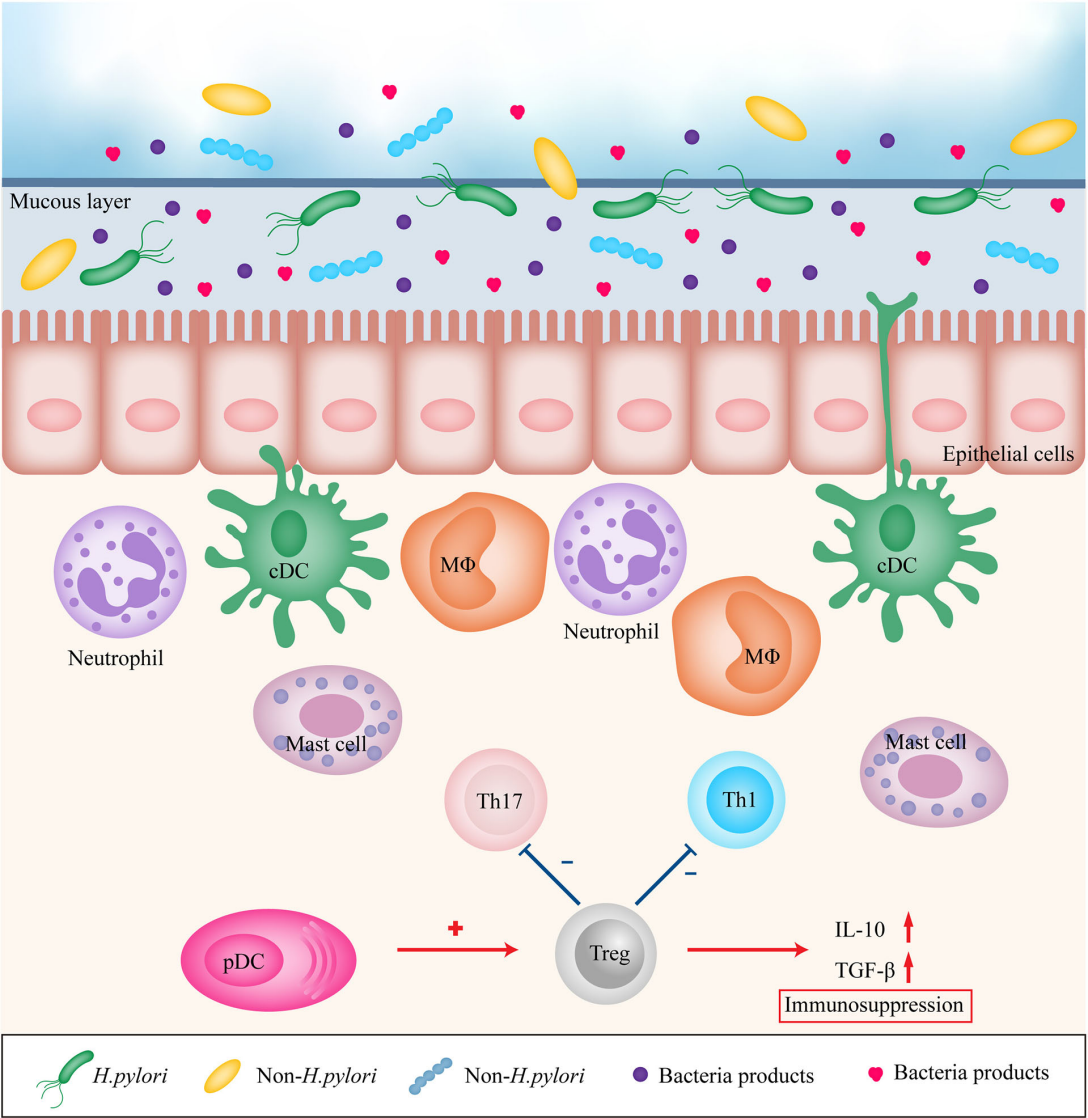
Immune modulation in gastric mucosa during inflammation. *H. pylori*, other bacteria and their products stimulates gastric epithelial cells and recruits immune cells to the site of infection, leading to persistent chronic inflammation. However, pDCs may recruit Treg cells to promote immunosuppression. Treg, regulatory T cell; Th1, T helper cell type 1; Th17, T helper cell type 17; DC, dendritic cell; MΦ, macrophage; pDC, plasmacytoid dendritic cell; IL-10, interleukin 10; TGF-β, transforming growth factor-β.

The gastrointestinal mucosa, which forms the main interface between the human host and its environment, resists attacks from microorganisms, their products, and other toxins. Therefore, the immune system serves an essential purpose to maintain the defense property. The gastric mucosa consists of an epithelial layer, lamina propria, and mucosal muscle layer, which together form not only a simple physical barrier but also complex chemical and biological barriers. Innate and adaptive immunity synergistically contribute to the homeostasis of the gastric mucosa. When the balance of mucosal immunity is disrupted, certain gastric diseases may occur. Inflammation is a well-recognized risk factor for cancer. *H. pylori*-induced chronic inflammation in the gastric mucosa is a key step in the initiation of the development of GC, and eradication of *H. pylori* infection is recommended to prevent GC (5). *H. pylori* stimulates gastric epithelial cells and recruits immune cells to the site of infection (6) (**Figure 1**). *H. pylori* infection is followed by upregulation of expression of various pro-inflammatory factors, including interleukin 1 (IL-1), IL-2, IL-6, IL-8, IL-12, tumor necrosis factor-α (TNF-α), and interferon γ (IFN-γ). Current evidence suggests that pro-inflammatory factors are critical participants in gastric carcinogenesis (7–9). However, the majority of *H. pylori* infected individuals do not develop GC (10) *H. pylori* infected patients with severe atrophy, corpus-predominant gastritis, and intestinal metaplasia are at high risk for intestinal-type GC, and patients with moderate atrophy and pangastritis are at high risk for diffuse-type GC, whereas isolated antral *H. pylori* infection or duodenal ulcer in *H. pylori* infected patients does not increase the risk of GC (11). Furthermore, British Society of Gastroenterology guidelines 2019 does not recommend surveillance in patients with gastric atrophy or gastric intestinal metaplasia limited just to the gastric antrum unless other risk factors are present (12).

Dendritic cells (DCs) are widely acknowledged as potent antigen-presenting cells (APCs), which are able to bridge innate and adaptive immunity. DCs can be classified into plasmacytoid DCs (pDCs) and conventional DCs (cDCs; also known as myeloid DCs). The pDCs have attracted increasing attention in recent years. Activated pDCs express various immunostimulatory and inhibitory molecules, secrete cytokines and chemokines, present antigens, and enhance the development and function of immune cells (13, 14). However, the precise roles of pDCs in gastric carcinogenesis and progression remain elusive. Here, we focus on current knowledge regarding immune modulation in GC, especially the role of pDCs in carcinogenesis and treatment strategies.

## 2 Immunobiology of pDCs

### 2.1 Characteristics of pDCs

Lennert and Remmele first described pDCs in 1958. They were found in the T cell zones of human lymph nodes and identified as T-associated plasma cells based on their morphological features (15). Over the past few decades, there have been significant new insights into pDCs. pDCs have a round morphology with an eccentric nucleus, harboring well-developed rough endoplasmic reticulum, Golgi apparatus, and many mitochondria (16), which account for about 0.1% of peripheral blood mononuclear cells (17). Human pDCs are phenotypically characterized by the presence of CD4, CD68, ILT3, IL-3 receptor α-subunit (IL-3R; also known as CD123), blood DC antigen 2 (BDCA-2; also known as CD303), CD304, and major histocompatibility class (MHC) II (18, 19).

pDCs originate from hematopoietic stem cells in the bone marrow (BM). pDCs can develop from IL-7R^+^ lymphoid precursor cells *via* the lymphoid pathway and are also derived from common DC progenitors *via* the myeloid pathway (20, 21). Fms-like tyrosine kinase 3 (Flt3) and its ligand (Flt3L) have critical roles in the developmental processes of pDCs (22–24). Flt3 and Flt3L activate signal transducer and activator of transcription 3 (STAT3) to promote the expression of the basic helix-loop-helix transcription factor E2-2/Tcf4, leading to the promotion of pDC development. Together, E2-2, E2a, and HEB make up the E protein family, which can form homo- or heterodimers to bind to cognate DNA sequences called E boxes (CANNTG), and subsequently activate or repress their target genes (25). E2-2 is essential for pDC development and the pDC-mediated IFN response in both mice and humans (26). However, granulocyte-macrophage colony-stimulating factor (GM-CSF) inhibits pDC development *via* STAT5-mediated expression of inhibitor of DNA binding 2 (ID2), which is an inhibitor of E2-2 (27).

Previously, pDCs were thought to be long-lived compared with cDCs based on the relatively slow rate of BrdU labeling by splenic pDCs (28, 29). However, parabiotic experiments in mice revealed that the turnover of pDC pools in the spleen and lymph nodes is more rapid than that of cDCs (30). Zhan et al. indicated that the slower rate of BrdU labeling of spleen pDCs may reflect the time required for pDCs labeled with BrdU to migrate to the spleen, rather than a longer lifespan of pDCs (31). The lifespan of pDCs is also short and is regulated by genetic and environmental factors (31). The underlying regulation mechanisms regarding the longevity of pDCs are not well understood, and further studies are needed.

### 2.2 Innate and Adaptive Immune Responses by pDCs

Distinct Toll-like receptors (TLRs) recognize different microbial components (32). TLR7 and TLR9 are key players in the sensing of pathogens by pDCs. Following ligand binding, the cytoplasmic Toll/IL-1 receptor (TIR) domains of TLR7 and TLR9 interact with signaling adaptor myeloid differentiation primary-response gene 88 (MyD88), triggering the activation of IRF7 to produce large amounts of type I IFNs (33, 34). IRF5 is critical for the induction of production of pro-inflammatory cytokines by the MyD88–nuclear factor kappa B (NF-κB) pathway (35). Type I IFNs comprise a large family of cytokines, consisting of IFN-α, IFN-β, IFN-ϵ, IFN-κ, and IFN-ω (36). It is widely accepted that type I IFNs have important roles in anti-viral immunity, both *in vitro* and *in vivo*. Type I IFNs also function as links between innate and adaptive immune responses. Type I IFNs can act directly or indirectly on multiple immune cells, including natural killer (NK) cells, T cells, B cells, DCs, and phagocytic cells (37–41).

Direct type I IFN signaling can play a critical part in clonal expansion of CD4^+^ and CD8^+^ T cells in certain infections (39, 42, 43). Moreover, type I IFNs can substitute T cells help *via* direct promotion of the survival and differentiation of CD8^+^ T cells during viral infections (44). pDCs can regulate the development of both T helper cell type 1 (Th1) and Th2 responses, depending on a variety of factors (45). pDCs stimulated with a virus and CD40L can efficiently drive Th1 cell polarization through the synergistic effects of IL-12 and type 1 IFNs (46). The virus converts the CD40L-mediated Th2 chemokine profile of pDCs into a potent Th1 mediator profile *via* the IFN-γ autocrine loop (47). Type 1 IFNs also limit the development of Th17 and negatively regulate Th17-mediated immune responses (48, 49). IFN-α inhibits spontaneous apoptosis of CD4^+^ and CD8^+^ T cells (50). It has been reported that pDCs regulate B cell responses *via* production of type 1 IFNs and IL-6, and by direct cell-to-cell contact (40). Data suggest that IFN-α secreted by pDCs makes B cells more responsive to T cell help and subsequently facilitates proliferation and differentiation of B cells with a T-cell-dependent pattern (51).

In addition to generating abundant amounts of type I IFNs, pDCs also act as APCs to activate adaptive immune responses. Owing to their expression of MHC molecules and costimulatory molecules CD40, CD80, and CD86, pDCs are thought to possess antigen presenting capacity (52). Activated pDCs have the potential to persistently synthesize, ubiquitinate, and turn over the MHC II–peptide complexes (41). This ability enables pDCs to consistently process and present endogenous self or viral antigens following activation, while the efficiency of presenting exogenous antigens is relatively low compared with those of cDCs (41).

In recent years, it has been suggested that pDCs can perform cross-presentation for efficient activation of T cells. Human circulating pDCs have similar ability to BDCA3^+^ cDCs to cross-present soluble and cell-associated tumor antigens to cytotoxic T cells, albeit pDCs take up less antigens than cDCs (53). Moreover, pDCs preserve exogenous antigens for a prolonged period of time and upregulate MHC I and II molecules after stimulation, which indicates the essential role of pDCs in cross-presenting extracellular antigens (53). Human pDCs can capture antigens in the form of microvesicles such as exosomes and apoptotic bodies (54). A recent study found that cross-presenting pDCs require pDC-derived exosomes to transfer antigens from pDCs to cDCs for cross-priming of naive CD8^+^ T cells (55).

Intriguingly, the ability of pDCs to induce peripheral tolerance in different situations has been well established; this function is predominantly realized through induction of regulatory T (Treg) cells (56–58). pDCs could cargo antigens to draining lymph nodes and transfer antigens to lymph node-resident APCs, leading to abortive proliferation of cognate CD4^+^ T cells and inducing tolerance (59). In addition, peripheral pDCs transport peripheral antigens in a central chemokine receptor 9-dependent manner to the thymus and subsequently delete the antigen-reactive thymocytes to promote immune tolerance (60). Recent studies have indicated that pDCs contribute to immune tolerance by expressing indoleamine 2,3-dioxygenase, inducible co-stimulator ligand (ICOS-L), OX40L, PD-L1, and granzyme B (61–65).

### 2.3 pDCs in GC

There are limited number of studies about the role of pDCs in GC (**Table 1**), and there is a deficiency of experiments (such as animal models of GC) to confirm the role of pDCs in GC. In peripheral blood, the increased numbers of pDCs, Treg cells, and ICOS^+^ Treg cells were found in GC patients when compared with healthy controls (66, 69). The enrichment of circulating pDCs was detected in GC patients with advanced stages and lymph node metastasis (69). Even though the number of circulating pDCs elevated, the plasma IFN-α level was decreased in GC patients (66). In breast cancer, the capacity of tumor-associated pDCs to produce type I IFN is substantially impaired, which in turn potentiates their capacity to promote the proliferation of tumor-associated forkhead box protein 3 (FOXP3)^+^ Treg cells (74). In tissue, the higher pDCs percentage was related to larger tumor size (67). BDCA2^+^ pDCs were homogeneously distributed in the GC tissue, but the number of BDCA2^+^ pDCs was significantly high in Epstein-Barr virus-associated gastric carcinoma (EBVaGC) as compared with EBV-negative GC (72). In EBVaGC, a high number of BDCA2^+^ pDCs was associated with diffuse histology and tumor invasion depth (72). Moreover, pDCs were mainly distributed in peritumor tissue, whereas Treg cells and ICOS^+^ Treg cells were mainly distributed in tumor tissue (66). The similar distribution pattern of pDCs has been reported in cervical carcinomas (75). There was a positive correlation between pDCs and ICOS^+^ Treg cells in peripheral blood and peritumor tissue of GC patients, suggesting that pDCs may promote the differentiation of naïve CD4^+^ T cells into ICOS^+^ Tregs (66). Both tumor and peritumor tissue had higher Foxp3^+^ICOS^+^/Foxp3^+^ cell ratio when compared with normal tissue (66). The number of ICOS^+^ Treg cells in tumor and peritumor tissue increased with the progress of tumor stage in patients (66). Foxp3^+^ICOS^+^ Treg cells utilize distinct cytokines, for example IL-10, and thereby contribute to immunosuppression (76). The level of IL-10 increased from normal tissue to tumor tissue, mirroring the distribution of ICOS^+^ Treg cells (66). Therefore, pDCs may recruit ICOS^+^ Treg cells to promote immunosuppression of GC. A prospective study analyzing blood samples from 41 GC patients and tissue samples from 87 GC patients indicated that ICOS^+^Foxp3^+^ Treg cells and pDCs could predict poor prognosis of GC (71). Wang et al. analyzed data from the Cancer Genome Atlas Stomach Adenocarcinoma (TCGA-STAD) cohort and GSE62254 cohort, suggested that the higher risk score demonstrated a significantly lower overall survival time, and revealed positive correlation between increased risk score and infiltration of pDCs in GC (TCGA-STAD: P < 0.001, R = 0.49; GSE62254: P < 0.001, R = 0.39) (73). In other tumors, the prognostic values of pDC and Treg cells have also been evaluated (77, 78).

**Table 1:** Studies on pDCs in patients with gastric cancer.

Author	Year	Sample	Results	References
Xiao-Mei Huang et al.	2014	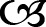 Peripheral blood 51 patients, 30 healthy individuals	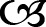 The numbers of pDCs, Tregs, and ICOS^+^ Tregs in peripheral blood were increased in GC patients compared with healthy donors.	(66)
		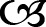 Tissue samples 91 patients	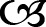 In tissue, Tregs and ICOS^+^ Tregs were found distributing mainly in carcinoma tissue, whereas pDCs were mainly found in peritumor tissue.	
			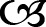 The Foxp3^+^ICOS^+^/Foxp3^+^ cell ratio in carcinoma and peritumor tissue were higher than that in normal tissue.	
			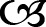 There were more ICOS^+^ Tregs in tumor and peritumor tissue of late-stage GC patients.	
			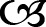 There was a positive correlation between pDCs and ICOS^+^ Tregs in peripheral blood and peritumor tissue from GC patients.	
Fangxuan Li et al.	2014	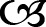 Tumor and normal tissues were obtained from 77 stomach cancer patients	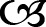 The higher pDCs percentage was associated with larger tumor size.	(67)
			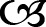 The ratio of myeloid DCs/pDCs was significantly lower in tumor tissues.	
Hirotsugu Nagase et al.	2016	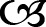 Peripheral blood was drawn from 40 GC patients and 5 healthy donors	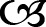 ICOS^+^ Foxp3^+^ CD4^+^ T cells were abundantly observed in the late stages of gastric cancer.	(68)
		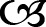 The surgically resected fresh cancer tissues of 40 GC, 10 colorectal cancers, 10 ovarian cancers, and 10 melanomas were obtained	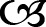 The expression of ICOS in Foxp3^+^ cells was closely related to pDCs and their expression of ICOS-L and TLR9 as well as *H. pylori* infection.	
Weihuang Liu et al.	2018	Peripheral blood samples		(69)
		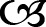 32 patients with GC	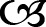 Patients with GC were identified to have substantially higher numbers of peripheral pDCs and mDC1s.	
		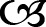 35 healthy volunteers	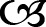 There was a trend of elevated circulating pDCs with advanced stages and lymph node metastasis in GC.	
Zongxin Ling et al.	2019	A cohort of 64 GC patients without preoperative chemotherapy was enrolled retrospectively, and 60 normal, 61 peritumoral and 59 tumoral tissues were obtained	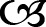 From different microhabitats, BDCA2^+^ pDCs and Foxp3^+^ Tregs were observed positively correlated, and increased in tumoral and peritumoral tissues compared to normal ones.	(70)
			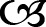 The diversity, composition and function of gastric mucosal microbiota also changed more significantly in tumoral tissues than those in normal and peritumoral ones.	
			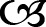 *Stenotrophomonas* and *Selenomonas* were positively correlated with BDCA2^+^ pDCs and Foxp3^+^ Tregs, respectively, while *Comamonas* and *Gaiella* were negatively correlated with BDCA2^+^ pDCs and Foxp3^+^ Tregs, respectively.	
Xiaosun Liu et al.	2019	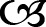 Peripheral blood from 41 GC patients	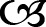 Both ICOS^+^Foxp3^+^ Treg cells and pDCs in peripheral blood and tumor tissue could predict poor clinical outcome in GC patients.	(71)
		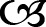 Carcinoma tissue, peritumor tissue and normal gastric mucosa from 87 GC patients		
Munetoshi Hinata et al.	2020	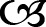 40 EBV-Negative GC	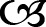 A high number of BDCA2^+^ DCs was correlated with tumor invasion depth and was inversely proportional to the number of CD1a-positive cells in EBVaGC.	(72)
		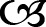 41 EBVaGC	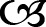 The ratio of BDCA2^+^ DCs to immature DCs was similarly low in the group with a high number of Langerhans- and CD1a-positive DCs.	
Zhenlin Wang et al.	2021	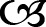 Samples from TCGA-STAD cohort and GSE62254 cohort	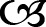 The infiltration of pDCs positively associated with GC prognostic index in the TCGA-STAD cohort.	(73)
		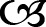 A total of 594 patients	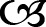 The infiltration of pDCs positively associated with GC prognostic index in the GSE62254 cohort.	

pDCs, plasmacytoid dendritic cells; ICOS, inducible co-stimulator; GC, gastric cancer; Foxp3, Forkhead box protein 3; DCs, dendritic cells; ICOS-L, inducible co-stimulator ligand; TLR9, Toll-like receptors 9; mDC1s, myeloid CD1c+ dendritic cells; BDCA2, blood DC antigen 2; EBV, Epstein-Barr virus; EBVaGC, EBV-associated gastric carcinoma; TCGA-STAD, the Cancer Genome Atlas Stomach Adenocarcinoma.

The human microbiome is essential for maintaining health, and a growing number of studies indicate a link between dysbiosis of the microbiome and diseases. Until the discovery of *H. pylori*, the stomach was long been thought to be sterile because of its highly acidic environment (79). As technology has developed, the mystery of the gastric microbiome has gradually been uncovered. The microbial load of the stomach is approximately 10^2^–10^4^ colony-forming units/ml (80). The most common phyla in gastric mucosa under normal conditions include *Proteobacteria*, *Firmicutes*, *Bacteroidetes*, *Actinobacteria*, and *Fusobacteria* (81). In healthy humans, the acidic gastric environment inhibits the over proliferation of microorganisms and reduces the risk of infection. *H. pylori* infection modulates the stomach environment. *H. pylori*-induced chronic inflammation triggers the loss of acid-secreting parietal cells, resulting in an increase in gastric pH, which promotes colonization by other bacteria (82). Although microbial dysbiosis has been observed during gastric carcinogenesis, there is currently no uniform pattern of alteration of the gastric microbiome. Alterations of the stomach microbiome promote the development of gastric diseases. Non-*H. pylori* bacteria promote gastric carcinogenesis by inducing inflammatory responses, modulating immune responses, triggering DNA damage, and promoting epithelial–mesenchymal transition (83–88).

As it is well known, *H. pylori* infection is associated with GC initiation, which leads to active inflammation and immune responses including the changes of pDCs. One study compared pDCs in *H. pylori*-infected children and noninfected controls and demonstrated upregulated expression of HLA-DR on circulating pDCs, and increased density of pDCs in gastric epithelium mucosa in *H. pylori*-infected children (89). Gastric mucosal microbiota analysis from 64 GC patients showed that the diversity, composition, and function of gastric mucosal microbiota was significantly different in tumor tissues compared with normal and peritumoral tissues, and BDCA2^+^ pDCs and Foxp3^+^ Tregs were positively correlated from different microhabitats (70). BDCA-2 is a novel type II C-type lectin that potently suppresses the production of IFN-α/β in pDCs (90). The changes in gastric mucosal microbiota and immune cells reflect the disturbance of the homeostasis of gastric mucosal immunity. In addition, *Stenotrophomonas* and *Selenomonas* were positively associated with BDCA2^+^ pDCs and Foxp3^+^ Tregs, respectively, whereas *Comamonas* and *Gaiella* were negatively associated with BDCA2^+^ pDCs and Foxp3^+^ Tregs, respectively (70). The gastric mucosal microbiota may play a part in modulating numbers of BDCA2^+^ pDCs and Foxp3^+^ Tregs to impair gastric mucosal immunity. Another study on the immune microenvironment of GC indicated close relationships among the expression of ICOS in Foxp3^+^ tumor-infiltrating lymphocytes and pDCs, the expression of ICOS-L and TLR9 of pDCs, and *H. pylori* infection (68).

However, the specific effects of microbiota on pDCs remains unclear. The commensal microbiota can shape systemic levels of pDCs *via* a novel mechanism involving cytolytic CD8^+^ T cells (91). Certain spherical lactic acid bacteria have various immunomodulatory effects on pDCs (92). When exposed to polysaccharide A of the gut commensal *Bacteroides fragilis*, pDCs stimulate IL-10 secretion by CD4^+^ T cells and mediate immunoprotection (93). One study monocolonized mice with single microbial strains derived from humans and found that the fluctuations of pDCs in monocolonized mice were bidirectional: 38% of the bacteria tested increased colonic pDC proportions, whereas 8% reduced colonic pDC proportions (94). Interestingly, the frequencies of pDCs were variable even in mice colonized by the same organism (94). One study compared the pDCs in germ-free and specific-pathogen-free mice and found that microbiota controlled trafficking and peripheral localization of pDCs by inducing sustained level of CCL2 (95).

## 3 Innate Immune Responses in GC

### 3.1 Role of the Innate Immune Receptors in GC

*H*. *pylori* is a Gram-negative bacterium with a spiral shape (96). The structural and functional characteristics of *H. pylori* confer it with the ability to resist acidic environments and colonize the host stomach (97). Persistent *H. pylori* infection initiates inflammatory responses of gastric mucosa, leading to atrophy of the glands, intestinal metaplasia, and GC (98). Immune responses to *H. pylori* infection are initiated by gastric epithelial cells and tissue-resident immune cells. The conserved pathogens’ molecules, such as lipopolysaccharide (LPS), double-stranded RNA, flagellin, and CpG repeats, is also known as pathogen-associated molecular patterns (PAMPs). Recognition of PAMPs of *H. pylori* by pattern recognition receptors (PRRs) triggers the initial stage of the host immune responses to *H. pylori*. PRRs are predominantly expressed in epithelial cells, DCs, macrophages, monocytes, and neutrophils. It has been reported that PRRs, including TLRs, nucleotide-binding oligomerization domain (NOD)-like receptors (NLRs), and retinoic acid-inducible gene I-like receptors, are involved in gastric carcinogenesis (99–101).

#### 3.1.1 TLRs in GC

As the major components of PRRs, TLRs play an essential part in *H. pylori* infection. Despite extensive study, the precise mechanism of TLRs is still controversial. TLR4 is the most studied TLR in *H. pylori* infection. TLR4 is activated by LPS of Gram-negative bacteria and subsequently triggers MyD88- and TIR-domain-containing adapter-inducing interferon-β-dependent signaling pathways, leading to the production of pro-inflammatory cytokines and type I IFNs (102). Accumulating evidence suggests that TLR2 has a significant role in recognition of *H. pylori* and induction of inflammatory responses. *H. pylori* infection upregulates the expression of TLR2 in gastric epithelial cells (103), whereas the expression pattern of TLR2 is not significantly altered after *H. pylori* eradication therapy (104). TLR9 mediates the recognition of *H pylori* DNA by DCs, inducing secretion of pro-inflammatory cytokines (105). Previous studies have demonstrated the high expression of TLR9 in GC (106, 107). The levels of *H. pylori*-induced TLR9 activation and expression are correlated with GC risk in different human populations (108). Nagase et al. hypothesized that chronic *H. pylori* infection might impact the expression of ICOS-L on pDCs *via* TLR9, leading to infiltration of ICOS^+^ Treg cells into GC (68). However, one study indicated that TLR9 had anti-inflammatory effects to suppress *H. pylori*-induced gastritis in the early phase of infection *via* reduction of Th1 response modulated by IFN-α (109). Taken together, TLR9 possesses both pro-inflammatory and anti-inflammatory effects, and more studies are needed to elucidate the detailed contributions of TLR9 in gastric carcinogenesis. In addition to TLR9, TLR7 is involved in recognition of purified *H. pylori* RNA, leading to the secretion of pro-inflammatory cytokines (110).

#### 3.1.2 NLRs in GC

In addition to TLRs, NLRs are involved in GC. NLRs are a type of intracellular PRRs, which can recognize not only PAMPs but also damage-associated molecular patterns (111). Much research has focused on the role during *H. pylori* infection of NOD1 and NOD2, which are expressed in gastric epithelial cells and APCs, where they recognize fragments of bacterial peptidoglycan (112). After recognizing γ-D-glutamyl-*meso*-diaminopimelic acid and muramyl dipeptide, respectively, NOD1 and NOD2 recruit receptor-interacting serine/threonine-protein kinase 2 through homotypic CARD–CARD interactions, resulting in the activation of NF-κB and mitogen-activated protein kinase pathways to induce robust pro-inflammatory responses (113–116).

### 3.2 Evasion From Recognition by PRRs

To survive, *H. pylori* employs multiple strategies to evade innate immune attack, including avoiding recognition by PRRs. LPS is a glycolipid present in the outer membrane of *H. pylori*, composed of lipid A, a core oligosaccharide, and O antigen (117). Compared with LPS of *Escherichia coli*, the LPS of *H. pylori* has lower immunological activity (118). During *H. pylori* infection, LPS modification contributes to persistent inflammation (117). Modification of the lipid A portion of LPS is used by *H. pylori* to escape immune recognition of TLR4 and evade the host innate immune response (119). Another example of evasion of recognition of PRRs is flagellin, which can be detected by TLR5 (120). *H. pylori* does not release flagellin, and the flagellin of *H. pylori* is less pro-inflammatory than that of *Salmonella typhimurium* (121).

### 3.3 Role of the Immune Cells in GC

#### 3.3.1 Macrophage

Macrophages are essential responders in innate immune responses and are among the most abundant tumor-infiltrating immune cells in solid tumors. Macrophages are classified into two groups, M1 and M2 macrophages. The classically activated M1 macrophages mediate pro-inflammatory effects. Activated M1 macrophages produce high levels of IL-1β, IL-6, IL-12, IFN-γ, TNF-α, CXCL9, and CXCL10 (122, 123). These molecules promotes the polarization and recruitment of Th1 cells to amplify type 1 responses and mediates the destruction of pathogens and tumor cells (124). By contrast, alternatively activated M2 macrophages impart anti-inflammatory properties by producing IL-4, IL-10, and transforming growth factor β1 (TGF-β1), which have pro-tumorigenic functions (125–127). Tumor-associated macrophages (TAMs) predominantly have M2 characteristics, which are correlated with poor prognosis in a variety of tumors (128, 129).

During *H. pylori* infection, macrophages are critical to the severity of gastric inflammation, possibly owing to cytokine secretion and/or antigen presentation (130). A single-cell gene expression study revealed that macrophages were enriched in GC tissue compared with normal tissue, and the gene expression profiles of macrophages were heterogenous, that is, they were not confined to a binary M1/M2 designation (131). Other bacteria in addition to *H. pylori* are involved in immune modulation in GC. The abundance of *Propionibacterium acnes* is significantly increased in GC tissues and is related to TNM stages of GC patients. *P. acnes* induces M2 polarization of macrophages through TLR4/PI3K/Akt signaling to promote progression of GC (132). The crosstalk between macrophages and GC cells or other immune cells contributes to shaping the immunosuppressive microenvironment and to GC progression (133–135). Furthermore, macrophages interact with mesenchymal stromal cells in GC. GC-derived mesenchymal stromal cells utilize cell-to-cell contact, paracrine effects, or extracellular vesicle transfer to induce the polarization of M2 macrophages, thereby prompting proliferation, invasion, and metastasis of GC (129, 136). A meta-analysis revealed that infiltration of M2 macrophages and total TAMs could be negative prognostic factors in GC, whereas M1 macrophage infiltration could be correlated with favorable survival rates (137). However, another study observed high infiltration of M2 macrophages in signet ring cell carcinoma and mucinous adenocarcinoma, which was associated with a favorable prognosis (138).

#### 3.3.2 Neutrophils

Neutrophils are an important component of the innate response and are the first responders to infection and inflammation. Neutrophils are enriched in GC tissue, and correlate with poor prognosis in GC (139, 140). Tumor-associated neutrophils (TANs) have been reported to participate in cancer initiation and progression *via* several mechanisms (141). GC-derived GM-CSF activates neutrophils and induce the expression of PD-L1 *via* Janus kinase-STAT3 signaling pathway, leading to the suppression of T cell function to promote GC progression (140). TANs also produce cytokines, such as IL-1β, IL-6, IL-8, IL-17a, and IL-23, to facilitate migration and invasion of GC (139, 142, 143).

#### 3.3.3 Mast Cells

Mast cells are another important BM-derived hematopoietic cells and widely distributed throughout the body (144). There are a substantial number of infiltrating mast cells in GC, which is correlated with tumor progression and predict shorter overall survival (145). Mast cells produce numerous mediators including pre-formed granule-associated mediators, newly generated lipid mediators, and a wide variety of cytokines and chemokines to exercise their biological functions (144). Tumor-derived TNF-α induces PD-L1 expression on intratumoral mast cells, which subsequently inhibits T cell function to suppress antitumor immunity in GC (145). Tumor-derived adrenomedullin activates mast cell degranulation, leading to the release of IL-17a to promote tumor progression (146). The mast cells-derived IL-17a also contributes to the tumor fibrosis in peritoneal dissemination in GC (147).

Taken together, the results of previous studies indicate both pro- and anti-oncogenic activities of immune cells in GC. However, the interaction between pDCs and other critical innate immune cells in GC still remains obscure, which is an important future research direction. Accumulating evidence suggests that tumor-infiltrating immune cells could be used to predict the prognosis of patients with GC. Although the molecular mechanism of immune cells in GC has not yet been fully elucidated, their potential therapeutic value is being investigated vigorously.

## 4 pDCs-Based Immunotherapy in Cancer

Traditional approaches for cancer treatment, including surgery, chemotherapy, and radiation therapy, are not always satisfactory. Therefore, effective therapies for cancer are urgently needed. In recent years, the close relationship between cancer and the immune system has attracted increasing attention. pDCs are not abundant in peripheral blood or in the tumor microenvironment, but they represent potential targets for cancer immunotherapy owing to their interactions with other immune cells.

The strategies to create antitumor immunity include pDC vaccination. DC-based vaccines have shown benefits in multiple tumor types. Vaccination of metastatic melanoma patients using naturally occurring pDCs has been confirmed to be safe and to induce antigen-specific CD4^+^ and CD8^+^ T-cell responses (148). Dey Mahua et al. compared the immune response generated by pDCs *vs*. cDCs in a DC-based vaccine strategy in a mouse glioma model; the results indicated that cDCs were more effective than pDCs in generating an anti-glioma Th-1 immune response (149). Nine patients with metastatic stage IV melanoma received cancer vaccines based on an allogeneic pDC line. Clinical observations demonstrated the capacity of the vaccines to prime and expand antitumor CD8^+^ responses, and no significant side effects were observed (150). In melanoma, vaccination with pDCs or CD1c^+^ DCs caused secretion of different chemokines and recruitment of different immune effector cells, in particular, pDCs induced a stronger influx of cytolytic lymphocytes than CD1c^+^ DCs (151). Combining the two DC subsets may enhance the antitumor efficacy of the vaccine owing to the chemoattractive properties of pDCs and the superior T cell priming properties of CD1c^+^ DCs (151). CD1c^+^ DCs and pDCs were shown to cross-activate each other and enhance NK-cell-mediated killing of an NK-resistant tumor cell line, suggesting that combining human blood DC subsets could further improve anticancer vaccine efficacy (152). Other vaccines have been used for anti-tumor therapy *via* activation of pDCs. One study investigated the effects of tumor cells infected with a measles virus vaccine on human pDCs; the results suggested that the vaccine induced immunogenic tumor cell death, as well as triggering pDC maturation, IFN-α production, and tumor antigen cross-presentation (153). The combination of an infectious but replication-deficient herpes simplex virus 1 vaccine strain with pDCs induced strong cytotoxic activity against tumor cells (154). The pDC vaccine has not been validated in a large population of patients; additional studies with large numbers of subjects are required in the future. However, the pDC vaccine is a promising option for cancer treatment.

The antitumor activities of type I IFNs have been well established (38). However, the efficacy of the IFN treatment is unsatisfactory in humans, and it causes frequent and severe adverse events (155). Therapies that are more effective have been developed over the past decades. Immunotherapies based on type I IFNs can selectively deliver IFN activity to targeted cells to reduce the toxic side effects caused by systemic IFN activity (156). It has been confirmed that the interactions between type I IFNs and DCs are critical to triggering antitumor responses (157, 158). As mentioned above, cancer patients show higher tumor infiltration of pDCs, impaired production of type I IFNs by pDCs, and enhanced capacity to promote Treg cell expansion. Therefore, re-activation of pDCs to trigger the production of type I IFNs is a potential therapeutic approach. TLR7 and TLR9 agonists have been used to activate tumor-associated pDCs and showed some clinical benefit (159–161). The combination of imiquimod, a TLR7 agonist, and GM-CSF gene-transduced tumor vaccines activates pDCs and enhances their immunologic antitumor effects (162). Conjugation of TLR7 agonist to GC antigen MG7-Ag tri-epitope performed antitumor effects, which effectively induced the secretion of TNF-α, IFN-γ, and IL-12, and enhanced antibody-dependent cell-mediated cytotoxicity and cytotoxic T lymphocyte activity (163). GC vaccines, which were synthesized by covalent attachment of TLR7 agonist with MG7-Ag tetra-epitope, combined with 5- fluorouracil chemotherapy decreased tumor sizes and increased long-term survival rates by improving T cell responses and decreasing myeloid-derived suppressor cells (MDSCs) (164). A study using mice bearing autochthonous gastric tumors or subcutaneous C26 tumors explored the mechanism of antitumor immunity of TLR9 ligand CpG. Although CpG treatment did not significantly reduce growth of autochthonous gastric tumors, the suppressive function of MDSCs was blocked. Subsequent work revealed that CpG application stimulated the production of IFN-α by pDCs, which induced the maturation of MDSCs and blocked MDSCs suppressivity (165).

Therefore, based on the immunological properties of pDCs, reactivation of pDCs and restoring the production of type I IFNs could improve the efficiency of cancer treatments.

## 5 Conclusions

Many questions concerning the tumor immune microenvironment still need to be resolved, especially the role of pDCs. pDCs have been reported to infiltrate into a variety of tumors, including GC, accompanied by impaired production of type I IFNs. Evidence suggests that pDCs have some therapeutic effects on cancers. However, further clinical research and experimental studies are required to determine their clinical value in cancer treatment.

## Author Contributions

ZL and JY designed the review and revised the manuscript. JY performed the literature search and wrote the manuscript. YC, XL, JZ, and FJ performed the literature search and analyzed the literature. ZL and JY prepared the manuscript figure and revised the manuscript. All authors contributed to the article and approved the submitted version.

## Funding

This present work was funded by the grants of the National Natural Science Foundation of China (81771724, 31700800, 81790631), the Research Project of Jinan Microecological Biomedicine Shandong Laboratory (JNL-2022033C), the Taishan Scholar Foundation of Shandong Province (tsqn202103119), the Nutrition and Care of Maternal & Child Research Fund Project of Guangzhou Biostime Institute of Nutrition & Care (2019BINCMCF045) and the National S&T Major Project of China (2018YFC2000500).

## Conflict of Interest

The authors declare that the research was conducted in the absence of any commercial or financial relationships that could be construed as a potential conflict of interest.

## Publisher’s Note

All claims expressed in this article are solely those of the authors and do not necessarily represent those of their affiliated organizations, or those of the publisher, the editors and the reviewers. Any product that may be evaluated in this article, or claim that may be made by its manufacturer, is not guaranteed or endorsed by the publisher.

## References

[1] Bray F Ferlay J Soerjomataram I Siegel RL Torre LA Jemal A. Global Cancer Statistics 2018: GLOBOCAN Estimates of Incidence and Mortality Worldwide for 36 Cancers in 185 Countries. CA Cancer J Clin (2018) 68:394–424. doi: 10.3322/caac.21492 30207593

[2] Miller KD Goding Sauer A Ortiz AP Fedewa SA Pinheiro PS Tortolero-Luna G. Cancer Statistics for Hispanics/Latinos, 2018. CA Cancer J Clin (2018) 68:425–45. doi: 10.3322/caac.21494 30285281

[3] Feng R-M Zong Y-N Cao S-M Xu R-H. Current Cancer Situation in China: Good or Bad News From the 2018 Global Cancer Statistics? Cancer Commun (London England) (2019) 39:22. doi: 10.1186/s40880-019-0368-6 PMC6487510 31030667

[4] Siegel R Naishadham D Jemal A. Cancer Statistics for Hispanics/Latinos, 2012. CA Cancer J Clin (2012) 62:283–98. doi: 10.3322/caac.21153 22987332

[5] Wong BC-Y Lam SK Wong WM Chen JS Zheng TT Feng RE. Helicobacter Pylori Eradication to Prevent Gastric Cancer in a High-Risk Region of ChinaA Randomized Controlled Trial. JAMA (2004) 291:187–94. doi: 10.1001/jama.291.2.187 14722144

[6] Torok AM Bouton AH Goldberg JB. Helicobacter Pylori Induces Interleukin-8 Secretion by Toll-Like Receptor 2- and Toll-Like Receptor 5-Dependent and -Independent Pathways. Infect Immun (2005) 73:1523–31. doi: 10.1128/IAI.73.3.1523-1531.2005 PMC1064968 15731050

[7] Wang J He W Liu J Nong L Wei Y Yang F. Association of IL-6 Polymorphisms With Gastric Cancer Risk: Evidences From a Meta-Analysis. Cytokine (2012) 59:176–83. doi: 10.1016/j.cyto.2012.03.032 22554382

[8] da Costa DM Neves-Filho EHC Alves MKS Rabenhorst SHB. Interleukin Polymorphisms and Differential Methylation Status in Gastric Cancer: An Association With Helicobacter Pylori Infection. Epigenomics (2013) 5:167–75. doi: 10.2217/epi.13.7 23566094

[9] Haghshenas MR Hosseini SV Mahmoudi M Saberi-Firozi M Farjadian S Ghaderi A. IL-18 Serum Level and IL-18 Promoter Gene Polymorphism in Iranian Patients With Gastrointestinal Cancers. J Gastroenterol Hepatol (2009) 24:1119–22. doi: 10.1111/j.1440-1746.2009.05791.x 19638090

[10] Mills JC Samuelson LC. Past Questions and Current Understanding About Gastric Cancer. Gastroenterology (2018) 155:939–44. doi: 10.1053/j.gastro.2018.06.044 PMC6174109 29964037

[11] Uemura N Okamoto S Yamamoto S Matsumura N Yamaguchi S Yamakido M. Helicobacter Pylori Infection and the Development of Gastric Cancer. N Engl J Med (2001) 345:784–9. doi: 10.1056/NEJMoa001999 11556297

[12] Banks M Graham D Jansen M Gotoda T Coda S di Pietro M. British Society of Gastroenterology Guidelines on the Diagnosis and Management of Patients at Risk of Gastric Adenocarcinoma. Gut (2019) 68:1545–75. doi: 10.1136/gutjnl-2018-318126 PMC6709778 31278206

[13] Tel J Smits EL Anguille S Joshi RN Figdor CG de Vries IJM. Human Plasmacytoid Dendritic Cells Are Equipped With Antigen-Presenting and Tumoricidal Capacities. Blood (2012) 120:3936–44. doi: 10.1182/blood-2012-06-435941 22966165

[14] Schuster P Thomann S Werner M Vollmer J Schmidt B. A Subset of Human Plasmacytoid Dendritic Cells Expresses CD8α Upon Exposure to Herpes Simplex Virus Type 1. Front Microbiol (2015) 6:557. doi: 10.3389/fmicb.2015.00557 26082771 PMC4451679

[15] Lennert K Remmele W. Karyometric Research on Lymph Node Cells in Man. I. Germinoblasts, Lymphoblasts & Lymphocytes. Acta Haematol (1958) 19:99–113. doi: 10.1159/000205419 13520253

[16] Liu YJ. IPC: Professional Type 1 Interferon-Producing Cells and Plasmacytoid Dendritic Cell Precursors. Annu Rev Immunol (2005) 23:275–306. doi: 10.1146/annurev.immunol.23.021704.115633 15771572

[17] Ueda Y Hagihara M Okamoto A Higuchi A Tanabe A Hirabayashi K. Frequencies of Dendritic Cells (Myeloid DC and Plasmacytoid DC) and Their Ratio Reduced in Pregnant Women: Comparison With Umbilical Cord Blood and Normal Healthy Adults. Hum Immunol (2003) 64:1144–51. doi: 10.1016/j.humimm.2003.08.342 14630396

[18] Alexandra-Chloé V Rahul S Gary R Siranush S Karthik S James F. Single-Cell RNA-Seq Reveals New Types of Human Blood Dendritic Cells, Monocytes, and Progenitors. Science (80 ) (2017) 356:eaah4573. doi: 10.1126/science.aah4573 PMC5775029 28428369

[19] Balan S Saxena M Bhardwaj N. Dendritic Cell Subsets and Locations. Int Rev Cell Mol Biol (2019) 348:1–68. doi: 10.1016/bs.ircmb.2019.07.004 31810551

[20] Herman JS Sagar Grün D. FateID Infers Cell Fate Bias in Multipotent Progenitors From Single-Cell RNA-Seq Data. Nat Methods (2018) 15:379–86. doi: 10.1038/nmeth.4662 29630061

[21] Rodrigues PF Alberti-Servera L Eremin A Grajales-Reyes GE Ivanek R Tussiwand R. Distinct Progenitor Lineages Contribute to the Heterogeneity of Plasmacytoid Dendritic Cells. Nat Immunol (2018) 19:711–22. doi: 10.1038/s41590-018-0136-9 PMC7614340 29925996

[22] Tussiwand R Onai N Mazzucchelli L Manz MG. Inhibition of Natural Type I IFN-Producing and Dendritic Cell Development by a Small Molecule Receptor Tyrosine Kinase Inhibitor With Flt3 Affinity. J Immunol (2005) 175:3674–80. doi: 10.4049/jimmunol.175.6.3674 16148112

[23] Waskow C Liu K Darrasse-Jèze G Guermonprez P Ginhoux F Merad M. The Receptor Tyrosine Kinase Flt3 Is Required for Dendritic Cell Development in Peripheral Lymphoid Tissues. Nat Immunol (2008) 9:676–83. doi: 10.1038/ni.1615 PMC2746085 18469816

[24] Karsunky H Merad M Cozzio A Weissman IL Manz MG. Flt3 Ligand Regulates Dendritic Cell Development From Flt3+ Lymphoid and Myeloid-Committed Progenitors to Flt3+ Dendritic Cells *In Vivo* . J Exp Med (2003) 198:305–13. doi: 10.1084/jem.20030323 PMC2194067 12874263

[25] Grajkowska LT Ceribelli M Lau CM Warren ME Tiniakou I Nakandakari Higa S. Isoform-Specific Expression and Feedback Regulation of E Protein TCF4 Control Dendritic Cell Lineage Specification. Immunity (2017) 46:65–77. doi: 10.1016/j.immuni.2016.11.006 27986456 PMC5243153

[26] Cisse B Caton ML Lehner M Maeda T Scheu S Locksley R. Transcription Factor E2-2 Is an Essential and Specific Regulator of Plasmacytoid Dendritic Cell Development. Cell (2008) 135:37–48. doi: 10.1016/j.cell.2008.09.016 18854153 PMC2631034

[27] Li HS Yang CY Nallaparaju KC Zhang H Liu Y-J Goldrath AW. The Signal Transducers STAT5 and STAT3 Control Expression of Id2 and E2-2 During Dendritic Cell Development. Blood (2012) 120:4363–73. doi: 10.1182/blood-2012-07-441311 PMC3507145 23033267

[28] O’Keeffe M Hochrein H Vremec D Caminschi I Miller JL Anders EM. Mouse Plasmacytoid Cells: Long-Lived Cells, Heterogeneous in Surface Phenotype and Function, That Differentiate Into CD8(+) Dendritic Cells Only After Microbial Stimulus. J Exp Med (2002) 196:1307–19. doi: 10.1084/jem.20021031 PMC2193989 12438422

[29] Chen M Huang L Shabier Z Wang J. Regulation of the Lifespan in Dendritic Cell Subsets. Mol Immunol (2007) 44:2558–65. doi: 10.1016/j.molimm.2006.12.020 PMC2851400 17267035

[30] Liu K Waskow C Liu X Yao K Hoh J Nussenzweig M. Origin of Dendritic Cells in Peripheral Lymphoid Organs of Mice. Nat Immunol (2007) 8:578–83. doi: 10.1038/ni1462 17450143

[31] Zhan Y Chow KV Soo P Xu Z Brady JL Lawlor KE. Plasmacytoid Dendritic Cells Are Short-Lived: Reappraising the Influence of Migration, Genetic Factors and Activation on Estimation of Lifespan. Sci Rep (2016) 6:25060. doi: 10.1038/srep25060 27112985 PMC4844974

[32] Akira S Takeda K. Toll-Like Receptor Signalling. Nat Rev Immunol (2004) 4:499–511. doi: 10.1038/nri1391 15229469

[33] Honda K Ohba Y Yanai H Negishi H Mizutani T Takaoka A. Spatiotemporal Regulation of MyD88-IRF-7 Signalling for Robust Type-I Interferon Induction. Nature (2005) 434:1035–40. doi: 10.1038/nature03547 15815647

[34] Lim K-H Staudt LM. Toll-Like Receptor Signaling. Cold Spring Harb Perspect Biol (2013) 5:a011247–a011247. doi: 10.1101/cshperspect.a011247 23284045 PMC3579400

[35] Mitchell D Chintala S Dey M. Plasmacytoid Dendritic Cell in Immunity and Cancer. J Neuroimmunol (2018) 322:63–73. doi: 10.1016/j.jneuroim.2018.06.012 30049538

[36] Fuertes MB Woo S-R Burnett B Fu Y-X Gajewski TF. Type I Interferon Response and Innate Immune Sensing of Cancer. Trends Immunol (2013) 34:67–73. doi: 10.1016/j.it.2012.10.004 23122052 PMC3565059

[37] Paolini R Bernardini G Molfetta R Santoni A. NK Cells and Interferons. Cytokine Growth Factor Rev (2015) 26:113–20. doi: 10.1016/j.cytogfr.2014.11.003 25443799

[38] Oh JH Kim MJ Choi SJ Ban YH Lee HK Shin E-C. Sustained Type I Interferon Reinforces NK Cell-Mediated Cancer Immunosurveillance During Chronic Virus Infection. Cancer Immunol Res (2019) 7:584–99. doi: 10.1158/2326-6066.CIR-18-0403 30808680

[39] Cervantes-Barragan L Lewis KL Firner S Thiel V Hugues S Reith W. Plasmacytoid Dendritic Cells Control T-Cell Response to Chronic Viral Infection. Proc Natl Acad Sci USA (2012) 109:3012–7. doi: 10.1073/pnas.1117359109 PMC3286988 22315415

[40] Ding C Cai Y Marroquin J Ildstad ST Yan J. Plasmacytoid Dendritic Cells Regulate Autoreactive B Cell Activation *via* Soluble Factors and in a Cell-to-Cell Contact Manner. J Immunol (2009) 183:7140–9. doi: 10.4049/jimmunol.0901175 PMC3351849 19890051

[41] Young LJ Wilson NS Schnorrer P Proietto A ten Broeke T Matsuki Y. Differential MHC Class II Synthesis and Ubiquitination Confers Distinct Antigen-Presenting Properties on Conventional and Plasmacytoid Dendritic Cells. Nat Immunol (2008) 9:1244–52. doi: 10.1038/ni.1665 18849989

[42] Havenar-Daughton C Kolumam GA Murali-Krishna K. Cutting Edge: The Direct Action of Type I IFN on CD4 T Cells Is Critical for Sustaining Clonal Expansion in Response to a Viral But Not a Bacterial Infection. J Immunol (2006) 176:3315–9. doi: 10.4049/jimmunol.176.6.3315 16517698

[43] Aichele P Unsoeld H Koschella M Schweier O Kalinke U Vucikuja S. Cutting Edge: CD8 T Cells Specific for Lymphocytic Choriomeningitis Virus Require Type I IFN Receptor for Clonal Expansion. J Immunol (2006) 176:4525–9. doi: 10.4049/jimmunol.176.8.4525 16585541

[44] Wiesel M Kratky W Oxenius A. Type I IFN Substitutes for T Cell Help During Viral Infections. J Immunol (2011) 186:754–63. doi: 10.4049/jimmunol.1003166 21160039

[45] Boonstra A Asselin-Paturel C Gilliet M Crain C Trinchieri G Liu Y-J. Flexibility of Mouse Classical and Plasmacytoid-Derived Dendritic Cells in Directing T Helper Type 1 and 2 Cell Development: Dependency on Antigen Dose and Differential Toll-Like Receptor Ligation. J Exp Med (2003) 197:101–9. doi: 10.1084/jem.20021908 PMC2193804 12515817

[46] Cella M Facchetti F Lanzavecchia A Colonna M. Plasmacytoid Dendritic Cells Activated by Influenza Virus and CD40L Drive a Potent TH1 Polarization. Nat Immunol (2000) 1:305–10. doi: 10.1038/79747 11017101

[47] Bendriss-Vermare N Burg S Kanzler H Chaperot L Duhen T de Bouteiller O. Virus Overrides the Propensity of Human CD40L-Activated Plasmacytoid Dendritic Cells To Produce Th2 Mediators Through Synergistic Induction of IFN-{Gamma} and Th1 Chemokine Production. J Leukoc Biol (2005) 78:954–66. doi: 10.1189/jlb.0704383 16081597

[48] Moschen AR Geiger S Krehan I Kaser A Tilg H. Interferon-Alpha Controls IL-17 Expression *In Vitro* and *In Vivo* . Immunobiology (2008) 213:779–87. doi: 10.1016/j.imbio.2008.07.022 18926293

[49] Zhang L Yuan S Cheng G Guo B. Type I IFN Promotes IL-10 Production From T Cells to Suppress Th17 Cells and Th17-Associated Autoimmune Inflammation. PloS One (2011) 6:e28432–2. doi: 10.1371/journal.pone.0028432 PMC3232207 22163016

[50] Rodriguez B Lederman MM Jiang W Bazdar DA Gàrate K Harding CV. Interferon-Alpha Differentially Rescues CD4 and CD8 T Cells From Apoptosis in HIV Infection. AIDS (2006) 20:1379–89. doi: 10.1097/01.aids.0000233571.51899.ab 16791012

[51] Gujer C Sandgren KJ Douagi I Adams WC Sundling C Smed-Sörensen A. IFN-α Produced by Human Plasmacytoid Dendritic Cells Enhances T Cell-Dependent Naïve B Cell Differentiation. J Leukoc Biol (2011) 89:811–21. doi: 10.1189/jlb.0810460 PMC3100762 21233412

[52] Swiecki M Colonna M. The Multifaceted Biology of Plasmacytoid Dendritic Cells. Nat Rev Immunol (2015) 15:471–85. doi: 10.1038/nri3865 PMC4808588 26160613

[53] Tel J Schreibelt G Sittig SP Mathan TSM Buschow SI Cruz LJ. Human Plasmacytoid Dendritic Cells Efficiently Cross-Present Exogenous Ags to CD8+ T Cells Despite Lower Ag Uptake Than Myeloid Dendritic Cell Subsets. Blood (2013) 121:459–67. doi: 10.1182/blood-2012-06-435644 23212525

[54] Bastos-Amador P Pérez-Cabezas B Izquierdo-Useros N Puertas MC Martinez-Picado J Pujol-Borrell R. Capture of Cell-Derived Microvesicles (Exosomes and Apoptotic Bodies) by Human Plasmacytoid Dendritic Cells. J Leukoc Biol (2012) 91:751–8. doi: 10.1189/jlb.0111054 22319103

[55] Fu C Peng P Loschko J Feng L Pham P Cui W. Plasmacytoid Dendritic Cells Cross-Prime Naive CD8 T Cells by Transferring Antigen to Conventional Dendritic Cells Through Exosomes. Proc Natl Acad Sci USA (2020) 117:23730–41. doi: 10.1073/pnas.2002345117 PMC7519282 32879009

[56] de Heer HJ Hammad H Soullié T Hijdra D Vos N Willart MAM. Essential Role of Lung Plasmacytoid Dendritic Cells in Preventing Asthmatic Reactions to Harmless Inhaled Antigen. J Exp Med (2004) 200:89–98. doi: 10.1084/jem.20040035 15238608 PMC2213319

[57] Ochando JC Homma C Yang Y Hidalgo A Garin A Tacke F. Alloantigen-Presenting Plasmacytoid Dendritic Cells Mediate Tolerance to Vascularized Grafts. Nat Immunol (2006) 7:652–62. doi: 10.1038/ni1333 16633346

[58] Uto T Takagi H Fukaya T Nasu J Fukui T Miyanaga N. Critical Role of Plasmacytoid Dendritic Cells in Induction of Oral Tolerance. J Allergy Clin Immunol (2018) 141:2156–67.e9. doi: 10.1016/j.jaci.2017.11.048 29477579

[59] Kohli K Janssen A Förster R. Plasmacytoid Dendritic Cells Induce Tolerance Predominantly by Cargoing Antigen to Lymph Nodes. Eur J Immunol (2016) 46:2659–68. doi: 10.1002/eji.201646359 PMC5129535 27592607

[60] Hadeiba H Lahl K Edalati A Oderup C Habtezion A Pachynski R. Plasmacytoid Dendritic Cells Transport Peripheral Antigens to the Thymus to Promote Central Tolerance. Immunity (2012) 36:438–50. doi: 10.1016/j.immuni.2012.01.017 PMC3315699 22444632

[61] Pallotta MT Orabona C Volpi C Vacca C Belladonna ML Bianchi R. Indoleamine 2,3-Dioxygenase Is a Signaling Protein in Long-Term Tolerance by Dendritic Cells. Nat Immunol (2011) 12:870–8. doi: 10.1038/ni.2077 21804557

[62] Pedroza-Gonzalez A Zhou G Vargas-Mendez E Boor PP Mancham S Verhoef C. Tumor-Infiltrating Plasmacytoid Dendritic Cells Promote Immunosuppression by Tr1 Cells in Human Liver Tumors. Oncoimmunology (2015) 4:e1008355. doi: 10.1080/2162402X.2015.1008355 26155417 PMC4485712

[63] Diana J Griseri T Lagaye S Beaudoin L Autrusseau E Gautron A-S. NKT Cell-Plasmacytoid Dendritic Cell Cooperation *via* OX40 Controls Viral Infection in a Tissue-Specific Manner. Immunity (2009) 30:289–99. doi: 10.1016/j.immuni.2008.12.017 19217323

[64] Nakano R Yoshida O Kimura S Nakao T Yokota S Ono Y. Donor Plasmacytoid Dendritic Cells Modulate Effector and Regulatory T Cell Responses In Mouse Spontaneous Liver Transplant Tolerance. Am J Transplant Off J Am Soc Transplant Am Soc Transpl Surg (2021) 21:2040–55. doi: 10.1111/ajt.16412 PMC8628164 33247989

[65] Jahrsdörfer B Vollmer A Blackwell SE Maier J Sontheimer K Beyer T. Granzyme B Produced by Human Plasmacytoid Dendritic Cells Suppresses T-Cell Expansion. Blood (2010) 115:1156–65. doi: 10.1182/blood-2009-07-235382 PMC2920226 19965634

[66] Huang X-M Liu X-S Lin X-K Yu H Sun J-Y Liu X-K. Role of Plasmacytoid Dendritic Cells and Inducible Costimulator-Positive Regulatory T Cells in the Immunosuppression Microenvironment of Gastric Cancer. Cancer Sci (2014) 105:150–8. doi: 10.1111/cas.12327 PMC4317822 24261990

[67] Li F Huang J Li S Li H Yu J Ren X. The Subsets of Dendritic Cells and Memory T Cells Correspond to Indoleamine 2,3-Dioxygenase in Stomach Tumor Microenvironment. Tumor Biol (2014) 35:8691–8. doi: 10.1007/s13277-014-2126-3 24870595

[68] Nagase H Takeoka T Urakawa S Morimoto-Okazawa A Kawashima A Iwahori K. ICOS+ Foxp3+ TILs in Gastric Cancer Are Prognostic Markers and Effector Regulatory T Cells Associated With Helicobacter Pylori. Int J Cancer (2017) 140:686–95. doi: 10.1002/ijc.30475 27756099

[69] Liu W Zhao J Li Q Wang Q Zhou Y Tong Z. Gastric Cancer Patients Have Elevated Plasmacytoid and CD1c(+) Dendritic Cells in the Peripheral Blood. Oncol Lett (2018) 15:5087–92. doi: 10.3892/ol.2018.7990 PMC5840537 29552142

[70] Ling Z Shao L Liu X Cheng Y Yan C Mei Y. Regulatory T Cells and Plasmacytoid Dendritic Cells Within the Tumor Microenvironment in Gastric Cancer Are Correlated With Gastric Microbiota Dysbiosis: A Preliminary Study. Front Immunol (2019) 10:533. doi: 10.3389/fimmu.2019.00533 30936882 PMC6433099

[71] Liu X Yu H Yan C Mei Y Lin C Hong Y. Plasmacytoid Dendritic Cells and ICOS+ Regulatory T Cells Predict Poor Prognosis in Gastric Cancer: A Pilot Study. J Cancer (2019) 10:6711–5. doi: 10.7150/jca.34826 PMC6856898 31777600

[72] Hinata M Kunita A Abe H Morishita Y Sakuma K Yamashita H. Exosomes of Epstein-Barr Virus-Associated Gastric Carcinoma Suppress Dendritic Cell Maturation. Microorganisms (2020) 8:1776. doi: 10.3390/microorganisms8111776 PMC7697542 33198173

[73] Wang Z Wang Z Hu X Han Q Chen K Pang G. Extracellular Matrix-Associated Pathways Promote the Progression of Gastric Cancer by Impacting the Dendritic Cell Axis. Int J Gen Med (2021) 14:6725–39. doi: 10.2147/IJGM.S334245 PMC8520888 34675633

[74] Sisirak V Faget J Vey N Blay J-Y Ménétrier-Caux C Caux C. Plasmacytoid Dendritic Cells Deficient in Ifnα Production Promote the Amplification of FOXP3(+) Regulatory T Cells and Are Associated With Poor Prognosis in Breast Cancer Patients. Oncoimmunology (2013) 2:e22338–8. doi: 10.4161/onci.22338 PMC3583914 23482834

[75] Bontkes HJ Ruizendaal JJ Kramer D Meijer CJLM Hooijberg E. Plasmacytoid Dendritic Cells Are Present in Cervical Carcinoma and Become Activated by Human Papillomavirus Type 16 Virus-Like Particles. Gynecol Oncol (2005) 96:897–901. doi: 10.1016/j.ygyno.2004.10.040 15721448

[76] Ito T Hanabuchi S Wang Y-H Park WR Arima K Bover L. Two Functional Subsets of FOXP3+ Regulatory T Cells in Human Thymus and Periphery. Immunity (2008) 28:870–80. doi: 10.1016/j.immuni.2008.03.018 PMC2709453 18513999

[77] Labidi-Galy SI Sisirak V Meeus P Gobert M Treilleux I Bajard A. Quantitative and Functional Alterations of Plasmacytoid Dendritic Cells Contribute to Immune Tolerance in Ovarian Cancer. Cancer Res (2011) 71:5423 LP – 5434. doi: 10.1158/0008-5472.CAN-11-0367 21697280

[78] Jensen TO Schmidt H Møller HJ Donskov F Høyer M Sjoegren P. Intratumoral Neutrophils and Plasmacytoid Dendritic Cells Indicate Poor Prognosis and Are Associated With Pstat3 Expression in AJCC Stage I/II Melanoma. Cancer (2012) 118:2476–85. doi: 10.1002/cncr.26511 21953023

[79] Marshall BJ Warren JR. Unidentified Curved Bacilli in the Stomach of Patients With Gastritis and Peptic Ulceration. Lancet (1984) 323:1311–5. doi: 10.1016/S0140-6736(84)91816-6 6145023

[80] Delgado S Cabrera-Rubio R Mira A Suárez A Mayo B. Microbiological Survey of the Human Gastric Ecosystem Using Culturing and Pyrosequencing Methods. Microb Ecol (2013) 65:763–72. doi: 10.1007/s00248-013-0192-5 23397369

[81] Liu X Shao L Liu X Ji F Mei Y Cheng Y. Alterations of Gastric Mucosal Microbiota Across Different Stomach Microhabitats in a Cohort of 276 Patients With Gastric Cancer. EBioMedicine (2019) 40:336–48. doi: 10.1016/j.ebiom.2018.12.034 PMC6412016 30584008

[82] Plottel CS Blaser MJ. Microbiome and Malignancy. Cell Host Microbe (2011) 10:324–35. doi: 10.1016/j.chom.2011.10.003 PMC3264051 22018233

[83] van Baarlen P Troost FJ van Hemert S van der Meer C de Vos WM de Groot PJ. Differential NF-kappaB Pathways Induction by Lactobacillus Plantarum in the Duodenum of Healthy Humans Correlating With Immune Tolerance. Proc Natl Acad Sci USA (2009) 106:2371–6. doi: 10.1073/pnas.0809919106 PMC2650163 19190178

[84] Gur C Ibrahim Y Isaacson B Yamin R Abed J Gamliel M. Binding of the Fap2 Protein of Fusobacterium Nucleatum to Human Inhibitory Receptor TIGIT Protects Tumors From Immune Cell Attack. Immunity (2015) 42:344–55. doi: 10.1016/j.immuni.2015.01.010 PMC4361732 25680274

[85] Montalban-Arques A Wurm P Trajanoski S Schauer S Kienesberger S Halwachs B. Propionibacterium Acnes Overabundance and Natural Killer Group 2 Member D System Activation in Corpus-Dominant Lymphocytic Gastritis. J Pathol (2016) 240:425–36. doi: 10.1002/path.4782 PMC5111592 27538697

[86] Roscetto E Vitiello L Muoio R Soriano AA Iula VD Vollaro A. *In Vitro* Interaction of Stenotrophomonas Maltophilia With Human Monocyte-Derived Dendritic Cells. Front Microbiol (2015) 6:723. doi: 10.3389/fmicb.2015.00723 26236302 PMC4504169

[87] Rubinstein MR Wang X Liu W Hao Y Cai G Han YW. Fusobacterium Nucleatum Promotes Colorectal Carcinogenesis by Modulating E-Cadherin/β-Catenin Signaling *via* Its FadA Adhesin. Cell Host Microbe (2013) 14:195–206. doi: 10.1016/j.chom.2013.07.012 23954158 PMC3770529

[88] Koller VJ Marian B Stidl R Nersesyan A Winter H Simić T. Impact of Lactic Acid Bacteria on Oxidative DNA Damage in Human Derived Colon Cells. Food Chem Toxicol (2008) 46:1221–9. doi: 10.1016/j.fct.2007.09.005 17942208

[89] Helmin-Basa A Wiese-Szadkowska M Szaflarska-Popławska A Kłosowski M Januszewska M Bodnar M. Relationship Between Helicobacter Pylori Infection and Plasmacytoid and Myeloid Dendritic Cells in Peripheral Blood and Gastric Mucosa of Children. Mediators Inflamm (2019) 2019:7190596. doi: 10.1155/2019/7190596 31827378 PMC6885256

[90] Dzionek A Sohma Y Nagafune J Cella M Colonna M Facchetti F. BDCA-2, a Novel Plasmacytoid Dendritic Cell-Specific Type II C-Type Lectin, Mediates Antigen Capture and Is a Potent Inhibitor of Interferon Alpha/Beta Induction. J Exp Med (2001) 194:1823–34. doi: 10.1084/jem.194.12.1823 PMC2193584 11748283

[91] Fujiwara D Wei B Presley LL Brewer S McPherson M Lewinski MA. Systemic Control of Plasmacytoid Dendritic Cells by CD8+ T Cells and Commensal Microbiota. J Immunol (2008) 180:5843–52. doi: 10.4049/jimmunol.180.9.5843 PMC3439147 18424703

[92] Jounai K Ikado K Sugimura T Ano Y Braun J Fujiwara D. Spherical Lactic Acid Bacteria Activate Plasmacytoid Dendritic Cells Immunomodulatory Function *via* TLR9-Dependent Crosstalk With Myeloid Dendritic Cells. PloS One (2012) 7:e32588–8. doi: 10.1371/journal.pone.0032588 PMC3323594 22505996

[93] Dasgupta S Erturk-Hasdemir D Ochoa-Reparaz J Reinecker H-C Kasper DL. Plasmacytoid Dendritic Cells Mediate Anti-Inflammatory Responses to a Gut Commensal Molecule *via* Both Innate and Adaptive Mechanisms. Cell Host Microbe (2014) 15:413–23. doi: 10.1016/j.chom.2014.03.006 PMC4020153 24721570

[94] Geva-Zatorsky N Sefik E Kua L Pasman L Tan TG Ortiz-Lopez A. Mining the Human Gut Microbiota for Immunomodulatory Organisms. Cell (2017) 168:928–943.e11. doi: 10.1016/j.cell.2017.01.022 28215708 PMC7774263

[95] Swiecki M Miller HL Sesti-Costa R Cella M Gilfillan S Colonna M. Microbiota Induces Tonic CCL2 Systemic Levels That Control pDC Trafficking in Steady State. Mucosal Immunol (2017) 10:936–45. doi: 10.1038/mi.2016.99 PMC5423869 27827374

[96] Chmiela M Walczak N Rudnicka K. Helicobacter Pylori Outer Membrane Vesicles Involvement in the Infection Development and Helicobacter Pylori-Related Diseases. J BioMed Sci (2018) 25:78. doi: 10.1186/s12929-018-0480-y 30409143 PMC6225681

[97] Yoshiyama H Nakazawa T. Unique Mechanism of Helicobacter Pylori for Colonizing the Gastric Mucus. Microbes Infect (2000) 2:55–60. doi: 10.1016/s1286-4579(00)00285-9 10717541

[98] Correa P. Human Gastric Carcinogenesis: A Multistep and Multifactorial Process–First American Cancer Society Award Lecture on Cancer Epidemiology and Prevention. Cancer Res (1992) 52:6735–40. 1458460

[99] Liu YD Yu L Ying L Balic J Gao H Deng NT. Toll-Like Receptor 2 Regulates Metabolic Reprogramming in Gastric Cancer *via* Superoxide Dismutase 2. Int J Cancer (2019) 144:3056–69. doi: 10.1002/ijc.32060 PMC6590666 30536754

[100] Zhou K Song B Wei M Fang J Xu Y. MiR-145-5p Suppresses the Proliferation, Migration and Invasion of Gastric Cancer Epithelial Cells *via* the ANGPT2/NOD_LIKE_RECEPTOR Axis. Cancer Cell Int (2020) 20:416. doi: 10.1186/s12935-020-01483-6 32874130 PMC7456024

[101] Castaño-Rodríguez N Kaakoush NO Mitchell HM. Pattern-Recognition Receptors and Gastric Cancer. Front Immunol (2014) 5:336. doi: 10.3389/fimmu.2014.00336 25101079 PMC4105827

[102] Płóciennikowska A Hromada-Judycka A Borzęcka K Kwiatkowska K. Co-Operation of TLR4 and Raft Proteins in LPS-Induced Pro-Inflammatory Signaling. Cell Mol Life Sci (2015) 72:557–81. doi: 10.1007/s00018-014-1762-5 PMC4293489 25332099

[103] Li S Cao M Song L Qi P Chen C Wang X. The Contribution of Toll-Like Receptor 2 on Helicobacter Pylori Activation of the Nuclear Factor-Kappa B Signaling Pathway in Gastric Epithelial Cells. Microb Pathog (2016) 98:63–8. doi: 10.1016/j.micpath.2016.06.028 27364547

[104] Cadamuro ACT Rossi AFT Matos Biselli-Périco J Fucuta Pereira P Do Vale EPBM Acayaba R. Effect of Helicobacter Pylori Eradication on TLR2 and TLR4 Expression in Patients With Gastric Lesions. Mediators Inflamm (2015) 2015:481972. doi: 10.1155/2015/481972 25873761 PMC4385704

[105] Rad R Ballhorn W Voland P Eisenächer K Mages J Rad L. Extracellular and Intracellular Pattern Recognition Receptors Cooperate in the Recognition of Helicobacter Pylori. Gastroenterology (2009) 136:2247–57. doi: 10.1053/j.gastro.2009.02.066 19272387

[106] Fernandez-Garcia B Eiró N González-Reyes S González L Aguirre A González LO. Clinical Significance of Toll-Like Receptor 3, 4, and 9 in Gastric Cancer. J Immunother (2014) 37:77–83. doi: 10.1097/CJI.0000000000000016 24509170

[107] Wang TR Peng JC Qiao YQ Zhu MM Zhao D Shen J. Helicobacter Pylori Regulates TLR4 and TLR9 During Gastric Carcinogenesis. Int J Clin Exp Pathol (2014) 7:6950–5. PMC4230143 25400780

[108] Varga MG Shaffer CL Sierra JC Suarez G Piazuelo MB Whitaker ME. Pathogenic Helicobacter Pylori Strains Translocate DNA and Activate TLR9 *via* the Cancer-Associated Cag Type IV Secretion System. Oncogene (2016) 35:6262–9. doi: 10.1038/onc.2016.158 PMC5102820 27157617

[109] Otani K Tanigawa T Watanabe T Nadatani Y Sogawa M Yamagami H. Toll-Like Receptor 9 Signaling has Anti-Inflammatory Effects on the Early Phase of Helicobacter Pylori-Induced Gastritis. Biochem Biophys Res Commun (2012) 426:342–9. doi: 10.1016/j.bbrc.2012.08.080 22940550

[110] Meliț LE Mărginean CO Mărginean CD Mărginean MO. The Relationship Between Toll-Like Receptors and Helicobacter Pylori-Related Gastropathies: Still a Controversial Topic. J Immunol Res (2019) 2019:8197048. doi: 10.1155/2019/8197048 30863783 PMC6378784

[111] Schroder K Tschopp J. The Inflammasomes. Cell (2010) 140:821–32. doi: 10.1016/j.cell.2010.01.040 20303873

[112] Liu P Lu Z Liu L Li R Liang Z Shen M. NOD-Like Receptor Signaling in Inflammation-Associated Cancers: From Functions to Targeted Therapies. Phytomedicine (2019) 64:152925. doi: 10.1016/j.phymed.2019.152925 31465982

[113] Allison CC Kufer TA Kremmer E Kaparakis M Ferrero RL. Helicobacter Pylori Induces MAPK Phosphorylation and AP-1 Activation *via* a NOD1-Dependent Mechanism. J Immunol (2009) 183:8099–109. doi: 10.4049/jimmunol.0900664 20007577

[114] Caruso R Warner N Inohara N Núñez G. NOD1 and NOD2: Signaling, Host Defense, and Inflammatory Disease. Immunity (2014) 41:898–908. doi: 10.1016/j.immuni.2014.12.010 25526305 PMC4272446

[115] Girardin SE Boneca IG Viala J Chamaillard M Labigne A Thomas G. Nod2 Is a General Sensor of Peptidoglycan Through Muramyl Dipeptide (MDP) Detection. J Biol Chem (2003) 278:8869–72. doi: 10.1074/jbc.C200651200 12527755

[116] Chamaillard M Hashimoto M Horie Y Masumoto J Qiu S Saab L. An Essential Role for NOD1 in Host Recognition of Bacterial Peptidoglycan Containing Diaminopimelic Acid. Nat Immunol (2003) 4:702–7. doi: 10.1038/ni945 12796777

[117] Maldonado RF Sá-Correia I Valvano MA. Lipopolysaccharide Modification in Gram-Negative Bacteria During Chronic Infection. FEMS Microbiol Rev (2016) 40:480–93. doi: 10.1093/femsre/fuw007 PMC4931227 27075488

[118] Stead CM Beasley A Cotter RJ Trent MS. Deciphering the Unusual Acylation Pattern of Helicobacter Pylori Lipid A. J Bacteriol (2008) 190:7012–21. doi: 10.1128/JB.00667-08 PMC2580709 18757539

[119] Cullen TW Giles DK Wolf LN Ecobichon C Boneca IG Trent MS. Helicobacter Pylori Versus the Host: Remodeling of the Bacterial Outer Membrane Is Required for Survival in the Gastric Mucosa. PloS Pathog (2011) 7:e1002454. doi: 10.1371/journal.ppat.1002454 22216004 PMC3245313

[120] Kim J-H Namgung B Jeon YJ Song WS Lee J Kang SG. Helicobacter Pylori Flagellin: TLR5 Evasion and Fusion-Based Conversion Into a TLR5 Agonist. Biochem Biophys Res Commun (2018) 505:872–8. doi: 10.1016/j.bbrc.2018.09.179 30301528

[121] Gewirtz AT Yu Y Krishna US Israel DA Lyons SL Peek RMJ. Helicobacter Pylori Flagellin Evades Toll-Like Receptor 5-Mediated Innate Immunity. J Infect Dis (2004) 189:1914–20. doi: 10.1086/386289 15122529

[122] Tarique AA Logan J Thomas E Holt PG Sly PD Fantino E. Phenotypic, Functional, and Plasticity Features of Classical and Alternatively Activated Human Macrophages. Am J Respir Cell Mol Biol (2015) 53:676–88. doi: 10.1165/rcmb.2015-0012OC 25870903

[123] Engström A Erlandsson A Delbro D Wijkander J. Conditioned Media From Macrophages of M1, But Not M2 Phenotype, Inhibit the Proliferation of the Colon Cancer Cell Lines HT-29 and CACO-2. Int J Oncol (2014) 44:385–92. doi: 10.3892/ijo.2013.2203 PMC3898868 24296981

[124] Biswas SK Mantovani A. Macrophage Plasticity and Interaction With Lymphocyte Subsets: Cancer as a Paradigm. Nat Immunol (2010) 11:889–96. doi: 10.1038/ni.1937 20856220

[125] Tsuji Y Kuramochi M Golbar HM Izawa T Kuwamura M Yamate J. Acetaminophen-Induced Rat Hepatotoxicity Based on M1/M2-Macrophage Polarization, in Possible Relation to Damage-Associated Molecular Patterns and Autophagy. Int J Mol Sci (2020) 21:8998. doi: 10.3390/ijms21238998 PMC7730394 33256230

[126] Arango Duque G Descoteaux A. Macrophage Cytokines: Involvement in Immunity and Infectious Diseases. Front Immunol (2014) 5:491. doi: 10.3389/fimmu.2014.00491 25339958 PMC4188125

[127] Zhang L Li Z Ding G La X Yang P Li Z. GRP78 Plays an Integral Role in Tumor Cell Inflammation-Related Migration Induced by M2 Macrophages. Cell Signal (2017) 37:136–48. doi: 10.1016/j.cellsig.2017.06.008 28629783

[128] Kurahara H Shinchi H Mataki Y Maemura K Noma H Kubo F. Significance of M2-Polarized Tumor-Associated Macrophage in Pancreatic Cancer. J Surg Res (2011) 167:e211–9. doi: 10.1016/j.jss.2009.05.026 19765725

[129] Li W Zhang X Wu F Zhou Y Bao Z Li H. Gastric Cancer-Derived Mesenchymal Stromal Cells Trigger M2 Macrophage Polarization That Promotes Metastasis and EMT in Gastric Cancer. Cell Death Dis (2019) 10:918. doi: 10.1038/s41419-019-2131-y 31801938 PMC6892854

[130] Kaparakis M Walduck AK Price JD Pedersen JS van Rooijen N Pearse MJ. Macrophages Are Mediators of Gastritis in Acute Helicobacter Pylori Infection in C57BL/6 Mice. Infect Immun (2008) 76:2235–9. doi: 10.1128/IAI.01481-07 PMC2346689 18332213

[131] Sathe A Grimes SM Lau BT Chen J Suarez C Huang RJ. Single-Cell Genomic Characterization Reveals the Cellular Reprogramming of the Gastric Tumor Microenvironment. Clin Cancer Res (2020) 26:2640–53. doi: 10.1158/1078-0432.CCR-19-3231 PMC7269843 32060101

[132] Li Q Wu W Gong D Shang R Wang J Yu H. Propionibacterium Acnes Overabundance in Gastric Cancer Promote M2 Polarization of Macrophages *via* a TLR4/PI3K/Akt signaling. Gastric Cancer (2021) 24:1242–53. doi: 10.1007/s10120-021-01202-8 34076786

[133] Zhou Z Xia G Xiang Z Liu M Wei Z Yan J. A C-X-C Chemokine Receptor Type 2-Dominated Cross-Talk Between Tumor Cells and Macrophages Drives Gastric Cancer Metastasis. Clin Cancer Res An Off J Am Assoc Cancer Res (2019) 25:3317–28. doi: 10.1158/1078-0432.CCR-18-3567 PMC8955044 30796034

[134] Lin C He H Liu H Li R Chen Y Qi Y. Tumour-Associated Macrophages-Derived CXCL8 Determines Immune Evasion Through Autonomous PD-L1 Expression in Gastric Cancer. Gut (2019) 68:1764–73. doi: 10.1136/gutjnl-2018-316324 30661053

[135] Eissmann MF Dijkstra C Jarnicki A Phesse T Brunnberg J Poh AR. IL-33-Mediated Mast Cell Activation Promotes Gastric Cancer Through Macrophage Mobilization. Nat Commun (2019) 10:2735. doi: 10.1038/s41467-019-10676-1 31227713 PMC6588585

[136] Zheng P Li W. Crosstalk Between Mesenchymal Stromal Cells and Tumor-Associated Macrophages in Gastric Cancer. Front Oncol (2020) 10:571516. doi: 10.3389/fonc.2020.571516 33163402 PMC7581781

[137] Wang XL Jiang JT Wu CP. Prognostic Significance of Tumor-Associated Macrophage Infiltration in Gastric Cancer: A Meta-Analysis. Genet Mol Res (2016) 15(4):gmr15049040. doi: 10.4238/gmr15049040 27966749

[138] Liu X Xu D Huang C Guo Y Wang S Zhu C. Regulatory T Cells and M2 Macrophages Present Diverse Prognostic Value in Gastric Cancer Patients With Different Clinicopathologic Characteristics and Chemotherapy Strategies. J Transl Med (2019) 17:192. doi: 10.1186/s12967-019-1929-9 31174544 PMC6554965

[139] Li S Cong X Gao H Lan X Li Z Wang W. Tumor-Associated Neutrophils Induce EMT by IL-17a to Promote Migration and Invasion In Gastric Cancer Cells. J Exp Clin Cancer Res (2019) 38:6. doi: 10.1186/s13046-018-1003-0 30616627 PMC6323742

[140] Wang T-T Zhao Y-L Peng L-S Chen N Chen W Lv Y-P. Tumour-Activated Neutrophils in Gastric Cancer Foster Immune Suppression and Disease Progression Through GM-CSF-PD-L1 Pathway. Gut (2017) 66:1900–11. doi: 10.1136/gutjnl-2016-313075 PMC5739867 28274999

[141] Coffelt SB Wellenstein MD de Visser KE. Neutrophils in Cancer: Neutral No More. Nat Rev Cancer (2016) 16:431–46. doi: 10.1038/nrc.2016.52 27282249

[142] Mao Z Zhang J Shi Y Li W Shi H Ji R. CXCL5 Promotes Gastric Cancer Metastasis by Inducing Epithelial-Mesenchymal Transition and Activating Neutrophils. Oncogenesis (2020) 9:63. doi: 10.1038/s41389-020-00249-z 32632106 PMC7338464

[143] Zhang W Gu J Chen J Zhang P Ji R Qian H. Interaction With Neutrophils Promotes Gastric Cancer Cell Migration and Invasion by Inducing Epithelial-Mesenchymal Transition. Oncol Rep (2017) 38:2959–66. doi: 10.3892/or.2017.5942 28901479

[144] Marshall JS. Mast-Cell Responses to Pathogens. Nat Rev Immunol (2004) 4:787–99. doi: 10.1038/nri1460 15459670

[145] Lv Y Zhao Y Wang X Chen N Mao F Teng Y. Increased Intratumoral Mast Cells Foster Immune Suppression and Gastric Cancer Progression Through TNF-α-PD-L1 Pathway. J Immunother Cancer (2019) 7:54. doi: 10.1186/s40425-019-0530-3 30808413 PMC6390584

[146] Lv Y-P Peng L-S Wang Q-H Chen N Teng Y-S Wang T-T. Degranulation of Mast Cells Induced by Gastric Cancer-Derived Adrenomedullin Prompts Gastric Cancer Progression. Cell Death Dis (2018) 9:1034. doi: 10.1038/s41419-018-1100-1 30305610 PMC6180028

[147] Gunjigake K Kinoshita J Yamaguchi T Saito H Fujimori D Horiike T. Interleukin-17A Derived From Mast Cells Contributes to Fibrosis in Gastric Cancer With Peritoneal Dissemination. Gastric Cancer (2021) 24:31–44. doi: 10.1007/s10120-020-01092-2 32488650 PMC7790800

[148] Tel J Erik HJGA Baba T Schreibelt G Schulte BM Benitez-Ribas D. Natural Human Plasmacytoid Dendritic Cells Induce Antigen-Specific T-Cell Responses in Melanoma Patients. Cancer Res (2013) 73:1063–75. doi: 10.1158/0008-5472.CAN-12-2583 23345163

[149] Dey M Chang AL Miska J Wainwright DA Ahmed AU Balyasnikova IV. Dendritic Cell-Based Vaccines That Utilize Myeloid Rather Than Plasmacytoid Cells Offer a Superior Survival Advantage in Malignant Glioma. J Immunol (2015) 195:367–76. doi: 10.4049/jimmunol.1401607 PMC4475664 26026061

[150] Charles J Chaperot L Hannani D Bruder Costa J Templier I Trabelsi S. An Innovative Plasmacytoid Dendritic Cell Line-Based Cancer Vaccine Primes and Expands Antitumor T-Cells in Melanoma Patients in a First-in-Human Trial. Oncoimmunology (2020) 9:1738812. doi: 10.1080/2162402X.2020.1738812 32313721 PMC7153838

[151] van Beek JJP Flórez-Grau G Gorris MAJ Mathan TSM Schreibelt G Bol KF. Human pDCs Are Superior to Cdc2s in Attracting Cytolytic Lymphocytes in Melanoma Patients Receiving DC Vaccination. Cell Rep (2020) 30:1027–1038.e4. doi: 10.1016/j.celrep.2019.12.096 31995747

[152] van Beek JJP Gorris MAJ Sköld AE Hatipoglu I Van Acker HH Smits EL. Human Blood Myeloid and Plasmacytoid Dendritic Cells Cross Activate Each Other and Synergize in Inducing NK Cell Cytotoxicity. Oncoimmunology (2016) 5:e1227902–e1227902. doi: 10.1080/2162402X.2016.1227902 27853652 PMC5087293

[153] Guillerme J-B Boisgerault N Roulois D Ménager J Combredet C Tangy F. Measles Virus Vaccine-Infected Tumor Cells Induce Tumor Antigen Cross-Presentation by Human Plasmacytoid Dendritic Cells. Clin Cancer Res An Off J Am Assoc Cancer Res (2013) 19:1147–58. doi: 10.1158/1078-0432.CCR-12-2733 23339127

[154] Thomann S Boscheinen JB Vogel K Knipe DM DeLuca N Gross S. Combined Cytotoxic Activity of an Infectious, But Non-Replicative Herpes Simplex Virus Type 1 and Plasmacytoid Dendritic Cells Against Tumour Cells. Immunology (2015) 146:327–38. doi: 10.1111/imm.12509 PMC4582973 26194553

[155] Weber JS Yang JC Atkins MB Disis ML. Toxicities of Immunotherapy for the Practitioner. J Clin Oncol (2015) 33:2092–9. doi: 10.1200/JCO.2014.60.0379 PMC4881375 25918278

[156] Garcin G Paul F Staufenbiel M Bordat Y van der Heyden J Wilmes S. High Efficiency Cell-Specific Targeting of Cytokine Activity. Nat Commun (2014) 5:3016. doi: 10.1038/ncomms4016 24398568

[157] Diamond MS Kinder M Matsushita H Mashayekhi M Dunn GP Archambault JM. Type I Interferon Is Selectively Required by Dendritic Cells for Immune Rejection of Tumors. J Exp Med (2011) 208:1989–2003. doi: 10.1084/jem.20101158 21930769 PMC3182061

[158] Lorenzi S Mattei F Sistigu A Bracci L Spadaro F Sanchez M. Type I IFNs Control Antigen Retention and Survival of CD8α + Dendritic Cells After Uptake of Tumor Apoptotic Cells Leading to Cross-Priming. J Immunol (2011) 186:5142–50. doi: 10.4049/jimmunol.1004163 21441457

[159] Cheng Y Lemke-Miltner CD Wongpattaraworakul W Wang Z Chan CHF Salem AK. *In Situ* Immunization of a TLR9 Agonist Virus-Like Particle Enhances Anti-PD1 Therapy. J Immunother Cancer (2020) 8:e000940. doi: 10.1136/jitc-2020-000940 33060147 PMC7566437

[160] Angeles L. Warming “Cold” Melanoma With TLR9 Agonists. Cancer Discov (2018) 8:670. doi: 10.1158/2159-8290.CD-ND2018-004 29669722

[161] Teulings H-E Tjin EPM Willemsen KJ van der Kleij S Ter Meulen S Kemp EH. Anti-Melanoma Immunity and Local Regression of Cutaneous Metastases in Melanoma Patients Treated With Monobenzone and Imiquimod; a Phase 2 a Trial. Oncoimmunology (2018) 7:e1419113. doi: 10.1080/2162402X.2017.1419113 29632737 PMC5889200

[162] Narusawa M Inoue H Sakamoto C Matsumura Y Takahashi A Inoue T. TLR7 Ligand Augments GM-CSF-Initiated Antitumor Immunity Through Activation of Plasmacytoid Dendritic Cells. Cancer Immunol Res (2014) 2:568–80. doi: 10.1158/2326-6066.CIR-13-0143 24830413

[163] Wang X-D Gao N-N Diao Y-W Liu Y Gao D Li W. Conjugation of Toll-Like Receptor-7 Agonist to Gastric Cancer Antigen MG7-Ag Exerts Antitumor Effects. World J Gastroenterol (2015) 21:8052–60. doi: 10.3748/wjg.v21.i26.8052 PMC4499347 26185376

[164] Wang X Liu Y Diao Y Gao N Wan Y Zhong J. Gastric Cancer Vaccines Synthesized Using a TLR7 Agonist and Their Synergistic Antitumor Effects With 5-Fluorouracil. J Transl Med (2018) 16:120. doi: 10.1186/s12967-018-1501-z 29739434 PMC5941430

[165] Zoglmeier C Bauer H Nörenberg D Wedekind G Bittner P Sandholzer N. CpG Blocks Immunosuppression by Myeloid-Derived Suppressor Cells in Tumor-Bearing Mice. Clin Cancer Res (2011) 17:1765–75. doi: 10.1158/1078-0432.CCR-10-2672 21233400

